# Accurate Prediction of Open-Circuit Voltages of Lithium-Ion Batteries via Delta Learning

**DOI:** 10.1021/acs.jctc.5c00168

**Published:** 2025-05-15

**Authors:** Wai Yuet Chiu, Chongzhi Zhang, Rongzhi Gao, Ziyang Hu, GuanHua Chen

**Affiliations:** 1 Department of Chemistry, 25809 The University of Hong Kong, Pok Fu Lam Road, Hong Kong SAR 000000, China; 2 Hong Kong AI Lab Limited, Pak Shek Kok,Hong Kong SAR 000000, China

## Abstract

Accurate prediction of lithium-ion battery capacity before material synthesis is crucial for accelerating battery material discovery. The capacity can be theoretically determined by integrating open-circuit voltage vs state of charge (OCV-SoC) curves of electrode materials. OCV-SoC curves are traditionally computed using first-principles methods, either through geometry optimization (GO) with density functional theory (DFT) or molecular dynamics (MD) simulations of lithiation/delithiation processes using DFT or force fields. While MD simulations incorporate temperature effects that GO lacks, even DFT-based MD simulated OCV-SoC curves show systematic deviations from experimental results due to inherent approximations in DFT functionals. In this study, we performed MD simulations on 43 cathode materials to obtain their OCV-SoC curves. Initial results showed only moderate agreement with experimental data, yielding a coefficient of determination (*R* ^2^) of 0.249 and a mean absolute error (MAE) of 1.561 V. Considering the scarcity of data, we implemented a delta learning approach to calibrate the MD results without substantial computational overhead, achieving an improved *R* ^2^ of 0.933 and an MAE of 0.131 V on the testing set. This calibration method significantly enhanced the accuracy of energy density predictions, reducing the MAE from 106.0 to 10.7 Wh/kg. We also developed an automated delta learning platform to make this approach accessible to researchers without machine learning expertise.

## Graphical abstract

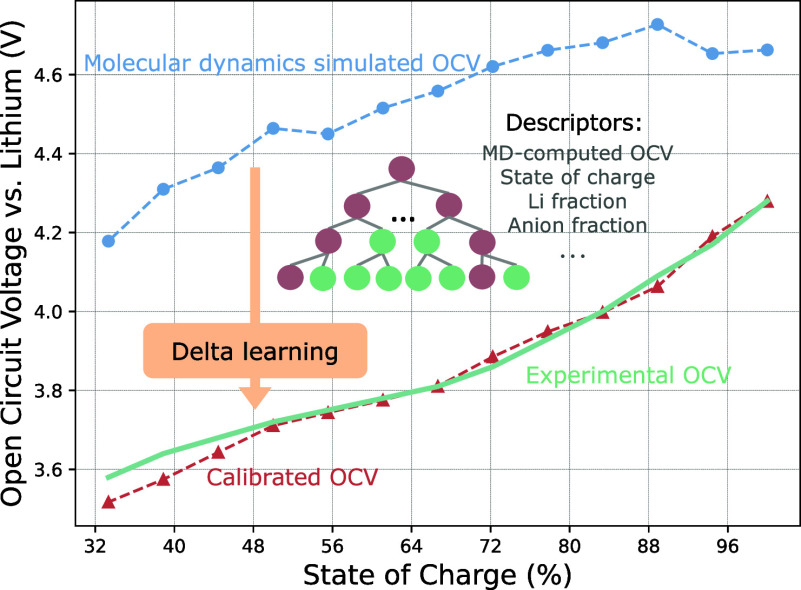

## 1 Introduction

Lithium-ion batteries stand out for their remarkably high specific energy density, typically ranging from 90 to 250 Wh/kg, far surpassing traditional battery technologies such as lead–acid and nickel–metal hydride.^1^ This superior energy density originates from lithium’s unique properties as the lightest metal, combined with the efficient intercalation mechanism. Recent advancements in electrode materials and cell design have pushed energy densities even higher, with some next-generation lithium-ion cells approaching 500 Wh/kg.^2^ First-principles calculations enable the rapid, cost-effective development of high-energy-density electrode materials by reducing the need for expensive experiments. Utilizing first-principles calculations like GO/MD with density functional theory (DFT),^3,4^ either internal energies or free energies of every state of charge during a lithiation or delithiation process can be obtained. The energy change per lithium atom is just an open-circuit voltage (OCV), and by integrating it across the state of charge range, the specific energy density is obtained. Through simulations over the vast possible chemical space of electrode materials, the discovery of high-energy materials will be expedited. For example, Zhang et al. utilized GO to simulate the regulation of voltage profiles of LiNiPO_4_ by doping,^5^ and Fu et al. applied MD to simulate the OCV-SoC curve of the silicon anode.^6^

While first-principles simulations offer qualitative insights, they may lack quantitative accuracy. Researchers face a dilemma between costly, precise computations and cheaper, less accurate alternatives. The delta learning method^7–9^ addresses this issue by calibrating low-fidelity data within machine learning frameworks to improve predictive accuracy. By leveraging both efficient machine learning models and the underlying physics of *ab initio* computations, the delta learning method achieves accurate results with reduced computational efforts. For example, it has been applied to improving DFT computed heats of formation to their experimental counterparts^10^ and prediction of the dopant formation energy for dopants in hafnia.^11^

This study applies delta learning to calibrate the OCV-SoC curves from molecular dynamics (MD) simulations against experimental data, effectively correcting systematic deviations in MD simulations. We also present an automated delta learning platform to help researchers obtain precise data cost-effectively, particularly for those without machine learning expertise. The paper is structured as follows: Section 2 examines results from both manual and automated delta learning, focusing on prediction accuracy and error distribution. Section 3 details the methodology, including data set construction, descriptor selection, and machine learning model development. Section 4 ends the article with perspectives.

## 2 Results and Discussion

### 2.1 Delta Learning with Manual Selection of Models

The delta learning model calibrates the low-fidelity MD simulated OCV values to their higher-fidelity experimental counterparts, by exploiting information provided by a set of descriptors.

Four widely used machine learning models were selected for prediction: random forest^12^ (RF), support vector regression^13^ (SVR), gradient boost^14^ (GB), and XGBoost.^15^ These commonly used models are chosen for delta learning applications due to their suitability for small data sets. These models incorporate intrinsic regularization mechanisms that effectively control the model complexity while preserving predictive capacity. Specifically, RF utilizes ensemble principles through decision tree aggregation, reducing variance without increasing bias. SVR implements kernel transformations that enable nonlinear relationship modeling with constrained parametrization. GB and XGBoost employ sequential weak learner construction with gradient-based optimization, focusing computational resources on residual errors from previous iterations. XGBoost additionally incorporates regularization terms and optimized tree construction algorithms that enhance sparsity handling and computational efficiency. The architectural characteristics of these models provide superior generalization capabilities under limited data conditions, making them particularly appropriate for delta learning scenarios, where data scarcity precludes the effective application of parameter-intensive deep learning approaches.

In the task of calibration, since MD-computed OCV is expected to introduce enough physics for estimating experimental OCV, simple machine learning models mentioned above will suffice for it. The effectiveness of calibration can be quantified by the coefficient of determination *R* ^2^ and the mean absolute error (MAE) between calculated and experimental OCVs, as shown in Table 1.

**Table 1:** Resulting Coefficient of Determination *R* ^2^ for Four Selected Machine Learning Models

**model**	**training** *R* ^**2**^	**training MAE (V)**	**testing** *R* ^**2**^	**testing MAE (V)**
MD (before calibration)	0.201	1.717	0.677	0.958
random forest	0.941	0.133	0.933	0.131
support vector regression	0.840	0.213	0.903	0.178
gradient boost	0.982	0.095	0.907	0.184
XGBoost	0.952	0.289	0.923	0.319

Before training, the MD simulated OCVs showed substantial deviations from experimental values, with MAEs of 1.717 V for the training set and 0.958 V for the testing set, yielding an overall MAE exceeding 1.56 V across all structures. While GB and XGBoost models performed best on the training set, they exhibited overfitting on the testing set. The RF model, showing consistent *R* ^2^ across both sets and superior testing set performance, was selected for further analysis. After RF calibration, the MAE improved dramatically to 0.133 V for the training set and 0.131 V for the testing set. The RF model effectively corrected the systematic deviation between simulated and experimental OCVs, as shown in Figure 1, where the predicted values closely align with the diagonal line. This alignment demonstrates successful calibration of MD-generated OCV-SoC curves to match experimental data, significantly improving the prediction accuracy and reliability.

**Figure 1:**
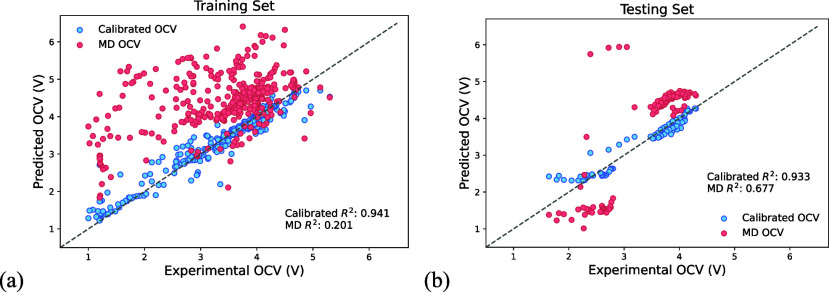
Random forest-calibrated results for both (a) training and (b) testing sets. The plots demonstrate the correlations between the input molecular dynamics (MD) OCV values and the calibrated OCV values. All the voltages are with respect to lithium. A systematic deviation exists between the MD OCVs and their experimental counterparts. The RF model corrected this deviation, resulting in predicted values that predominantly align with the diagonal line.

Figure 2 displays three representative RF calibrations from the testing set, highlighting how MD simulations consistently overestimated the OCV values through systematic deviations. While the RF model successfully calibrated these MD-generated OCV-SoC curves to match experimental data, as shown in Figure 1, its accuracy improves most when testing structures are similar to those in the training set. This is demonstrated by the three structures sharing the *R*3̅*m* symmetry group (space group 166), which differ only in composition and lattice parameters.

**Figure 2:**
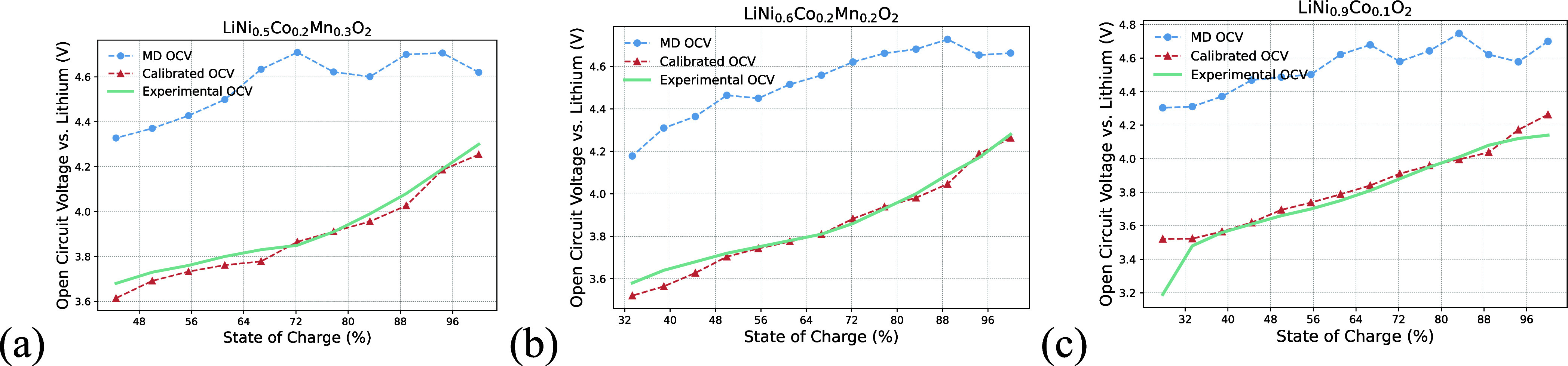
Comparison of molecular dynamics (MD) simulated, random forest (RF) calibrated, and experimental OCV-SoC curves for (a) LiNi_0.5_Co_0.2_Mn_0.3_O_2_,^16^ (b) LiNi_0.6_Co_0.2_Mn_0.2_O_2_,^17^ and (c) LiNi_0.9_Co_0.1_O_2_,^18^ demonstrating representative calibrations from the testing set. A systematic overestimation of the OCV calculated by molecular dynamics was clearly shown, which was diminished after random forest calibrations.

The strong correlation between prediction accuracy and structural similarity enables reliable battery energy density predictions, contingent on the model having encountered structurally similar materials. However, the current limited experimental data set lacks comprehensive structural diversity, potentially affecting prediction accuracy for novel structures. This limitation could be addressed by expanding the training data set to include more diverse structures and making the delta learning framework continuously upgradable.

### 2.2 Energy Density Prediction

OCV-SoC curve prediction serves as a valuable tool for battery material discovery by enabling theoretical capacity estimation before experimental synthesis and testing. The validation process compares energy densities derived from experimental and delta learning-calibrated OCV-SoC curves. Energy density calculations involve integrating the area under the curve to determine the total cell energy and then dividing by the mass of fully lithiated structures. The RF calibration significantly improves prediction accuracy, reducing the mean absolute error from 123.2 to 10.0 Wh/kg in the training set and from 106.0 to 10.7 Wh/kg in the testing set.

### 2.3 Error Analysis

Figure 3 compares error distributions in the testing set before and after delta learning calibration through histogram plotting error frequencies (in volts). The initial distribution shows substantial spread (−1.5 to 3 V) and positive skewness, confirming MD simulation’s tendency to overestimate OCV values. After delta learning calibration, the error distribution narrows significantly, demonstrating the effectiveness of the calibration process in improving the prediction accuracy.

**Figure 3:**
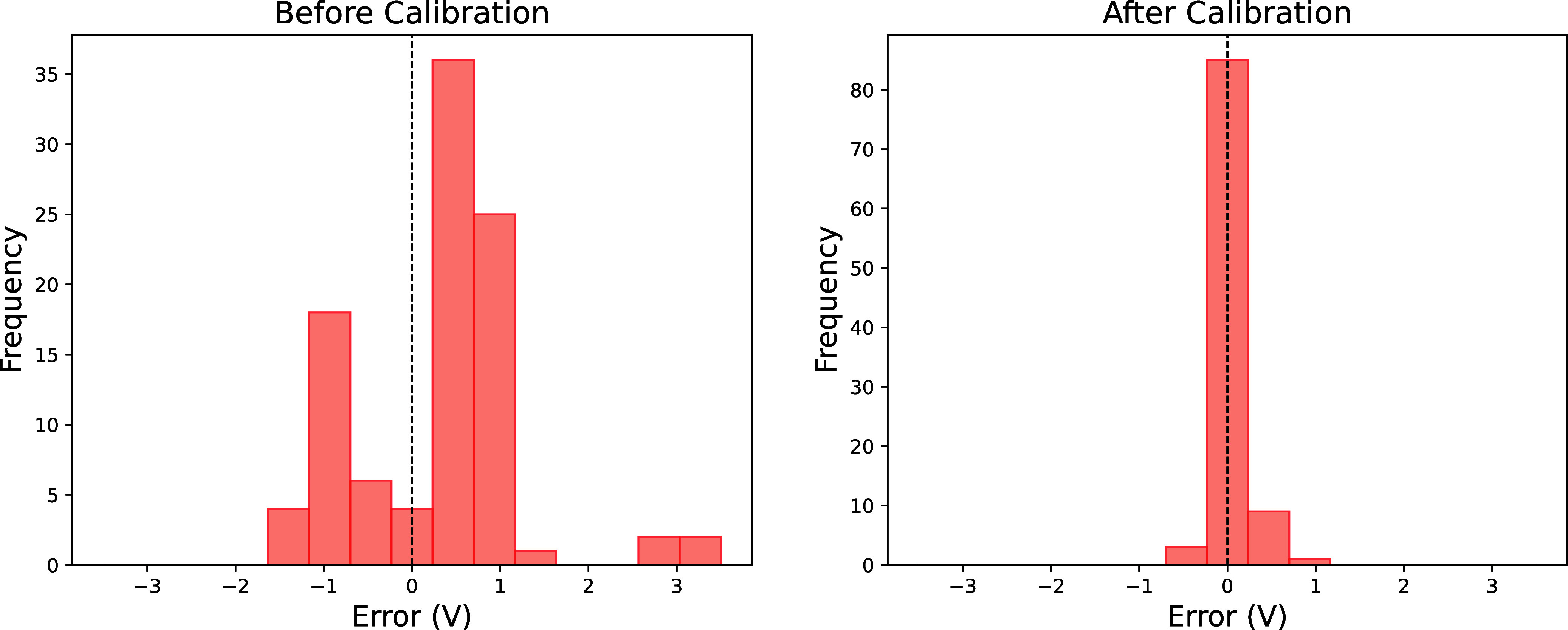
Histograms showing errors of the data set (left) before and (right) after delta learning. The errors were calculated as the differences between the predicted and experimental OCV values. The delta learning process effectively reduces the errors, resulting in a more accurate prediction of the OCV-SoC curves.

After calibration, the error histogram reveals a tighter distribution around zero, underscoring the effectiveness of the calibration process in aligning MD OCV values with experimental data. The reduction in both variance and bias of the errors indicates that the delta learning approach successfully mitigated discrepancies between simulated and experimental OCV curves, thereby enhancing the prediction fidelity.

### 2.4 Delta Learning with the Auto Delta Learning Platform

The auto delta learning platform is an innovative solution designed to make delta learning accessible to researchers without machine learning expertise. It simplifies the complexities typically associated with delta learning, allowing users to concentrate on their data and insights rather than on the underlying algorithms.

At the core of the platform is a user-friendly interface that guides users through the calibration process step by step. Users can easily upload their data sets, specifying their low-fidelity data alongside the corresponding high-fidelity targets. The platform automatically applies the necessary adjustments, including computing required descriptors, and refines model predictions by tuning hyperparameters. This automation significantly reduces the time and manual effort needed to achieve a high prediction accuracy, making it suitable for a wide range of applications across fields. Additionally, the platform provides versatile integration capabilities, allowing users to either implement their own custom predictive models or choose from a selection of preconfigured models optimized for specific applications.

For comparison, the same set of descriptors (listed in Table 2 in Section 3) used for manual training was fed into the platform, with the results summarized in Figure 4. The platform achieved *R* ^2^ of 0.980 for the training set and 0.878 for the testing set, with MAEs of 0.072 and 0.207 V, respectively. These results are comparable to those of manually trained models, highlighting the platform’s effectiveness.

**Figure 4:**
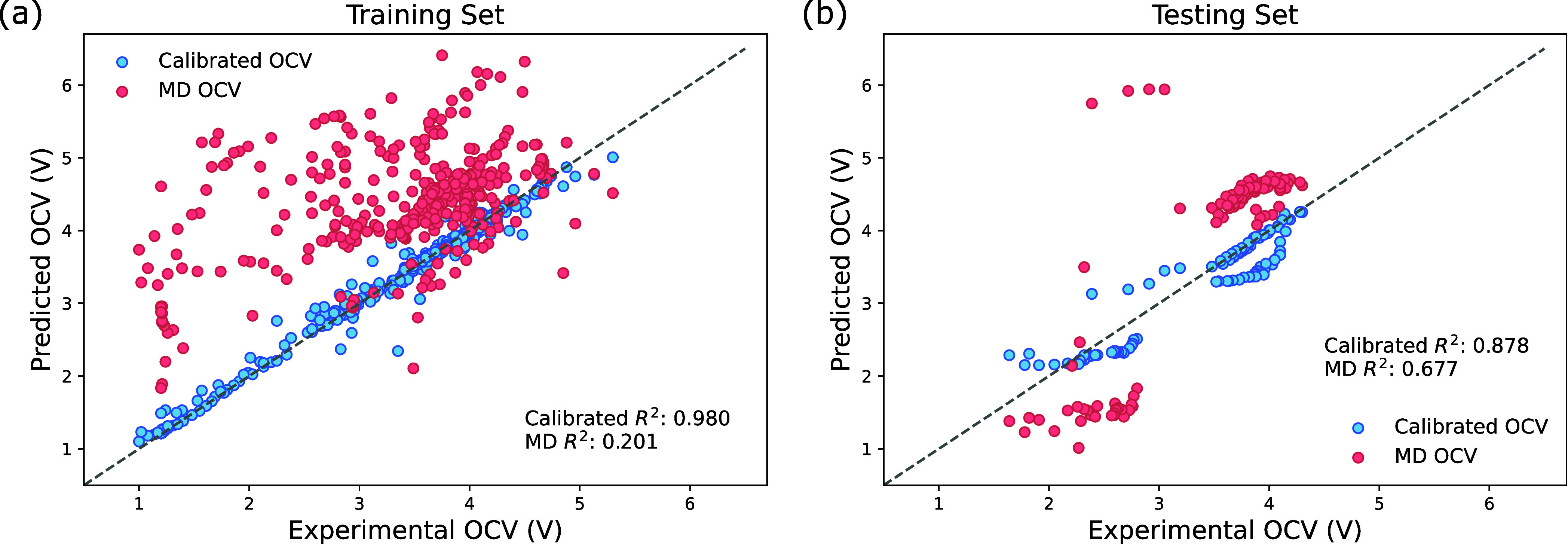
Results for (a) the training set and (b) the testing set from the model selected by the auto delta learning platform. The MAE for the training set is 0.072 V and 0.207 V for the testing set.

**Table 2:** List of Descriptors Used for Training Delta Learning Models, Selected with Chemistry Intuition^a^

**feature names**	**values**
MD-computed OCVs	change with positions along one OCV-SoC curve
SoC	
Li atomic fraction	
Li weight fraction	
mass per volume	remain the same within every OCV-SoC curve (computed from full-Li structures)
electron per volume	
TM atomic fraction	
TM weight fraction	
anion atomic fraction	
anion weight fraction	

a TM means transition metal here.

The auto delta learning platform offers the advantage of requiring less time and effort for tuning model hyperparameters, thus demanding less expertise from users. Still, the quality of the results is comparable to that achieved through manual training. This implies that the accuracy of predictions is mostly limited by the precision of the low-fidelity results and the appropriateness of the selected descriptors rather than the model architectures themselves.

## 3 Methods

### 3.1 Construction of the Data Set

The data set comprises 43 LIB cathode structures obtained from the Crystallography Open Database (COD),^19–21^ all validated through X-ray diffraction experiments. These structures and their corresponding OCV curves form 475 total data points. The data set is split with an 8:2 ratio randomly, allocating 34 structures (80%) for training the machine learning models and 9 structures (20%) for testing model performance. This structure-based data set from experimental literature provides the essential foundation for chemical modeling and subsequent analyses.

For each structure within the data set, molecular dynamics (MD) simulations were conducted to obtain OCV-SoC curves, which serve as low-fidelity data for subsequent delta learning calibration. X-ray diffraction structures are fully lithiated, and a set of delithiated structures is obtained by a special quasi-random (SQS) algorithm.^22^ The total possible configurations of every delithiated structure are tremendous, as calculated by the combination number *C*_*n*_^*m*^, where *n* and *m* are the number of the original lithium atoms and the number of lithium atoms to be removed, respectively. It is thus impossible to perform MD simulations on every single configuration. The SQS algorithm, which generates structures mimicking relevant radial correlation functions of a perfectly random structure, enables the estimation of thermodynamic properties of delithiated structures. For every delithiated structure, 20 SQS-generated structures are fully relaxed, after which the lowest-energy structure is selected to be the starting structure for MD simulations. (Further computational details are illustrated in Section B, Supporting Information). The randomness introduced in the SQS generation of structures will not affect the overall results (details shown in Section F, Supporting Information), with the MAE between two independent trials of the SQS-generated structure of different SoCs being 0.019 V, much lower than the training MAE of 0.148 V. The MD simulations were executed using a machine learning potential that accommodates polarizable long-range interactions,^23^ offering relatively low computational costs for large lattice structures. The machine learning potential is trained on 1,557,645 configurations from the Materials Project.^24^ The efficacy of machine learning in addressing delithiated structures is demonstrated in the Supporting Information. Gibbs free energies were computed by sampling the resulting trajectories using the method proposed by Lin et al.^25^ Further details on the construction of the data set can be found in Section A, Supporting Information.

### 3.2 Selection of Descriptors

Descriptor selection is a critical process in training machine learning models, as the model’s effectiveness largely depends on the quality and relevance of these descriptors. They should encapsulate the essential properties of materials, including structural and electronic characteristics, to enable the model to identify meaningful patterns and make accurate predictions.

In this work, the descriptors selected with chemical intuition are expected to resolve the systematic deviation between simulated and experimental OCVs and are of high importance to the target (shown in the Supporting Information). These descriptors are derived from the structures of the batteries, encompassing Li fractions, transition metal (TM) fractions, and anion fractions, as well as mass and electron densities. These descriptors incur minimal computational costs while yielding satisfactory prediction results.

Chemical intuition informs the selection of the descriptors listed in Table 2. The first kinds of descriptors, including MD OCV values, SoCs, Li atomic fractions, and Li weight fractions, indicate the positions of the OCVs along a given OCV-SoC curve, causing these features to vary along each curve. In contrast, the second kind of descriptors—mass per volume, electron density per volume, TM atomic and weight fractions, and anion (including all nonmetal atoms) atomic and weight fractions—represent properties of the fully lithiated structures. Consequently, these descriptors remain constant within a single OCV-SoC curve.

### 3.3 Construction of Machine Learning Models

All machine learning models were trained using sklearn in Python.^26^ The model parameters are summarized in Table 3 below.

**Table 3:** Hyper Parameters of the RF, SVR, GB, and XGBoost^a^

	model			
parameter	RF	SVR	GB	XGBoost
n_estimators	509	N/A	400	1000
max_depth	46	N/A	6	17
min_samples_split	2	N/A	7	2
min_samples_leaf	4	N/A	7	7
learning_rate	N/A	N/A	0.008	0.001
loss	squared_error	N/A	squared_error	squared_error
C	N/A	1000	N/A	N/A
degree	N/A	6	N/A	N/A
epsilon	N/A	3.16 × 10^–5^	N/A	N/A
gamma	N/A	1× 10^–6^	N/A	N/A
kernel	N/A	rbf	N/A	N/A

a The model parameters were optimized through Bayesian optimization. Details of the search spaces are listed in the Supporting Information.

### 3.4 Auto Delta Learning Platform

The auto delta learning platform leverages autosklearn, a Python package integrated with scikit-learn, to automate model training through Bayesian optimization, meta-learning, and ensemble construction.^27^ Users can upload a set of descriptors deemed suitable for the task based on expert intuition for model training. Alternatively, relevant features can be generated automatically. For instance, descriptors for small molecules can be computed from their SMILES strings using RDKit*,* ^28^ while descriptors for lattice structures involved in battery computations can be derived using matminer*.* ^29^ The platform employs a weighted ensemble of machine learning models, typically achieving better performance than single models. It also performs feature importance analysis, providing insights for descriptor optimization, regardless of whether the features were manually selected or automatically generated.

## 4 Conclusions

This study explores the application of delta learning for calibrating MD simulated OCV-SoC curves to their experimental counterparts. It demonstrates the effectiveness of delta learning as a calibration technique, highlighting its potential to enhance the accuracy of MD simulations in predicting battery performance. By employing machine learning models, we captured the intricate relationships between the simulated and experimental data, establishing a robust framework for improving the fidelity of MD simulations. Additionally, an automated delta learning platform can streamline this process, enabling rapid calibration without the need for extensive manual adjustments while achieving comparable accuracy in the OCV-SoC curve calibration. This automation reduces human error and time investment while improving the reproducibility and consistency in results.

While this study focused on the calibration of the OCV-SoC curves, the principles of delta learning can be applied to other areas of material science and electrochemistry. The delta learning approach presents a robust way for bridging simulation–experiment gaps in complex chemical systems, advancing predictive modeling capabilities in complex chemical environments.

## Supplementary Material



## Data Availability

The data that support the findings of this study are available from the authors upon reasonable request. The proof-of-concept code for the auto delta learning platform can be found at https://github.com/waiyuetchiu/delta_soc/blob/main/auto_delta.py, and the full platform can be provided to academic users upon request.

## Notes

Data source and more computational details, including machine learning interatomic potential, MD simulations, feature importance analysis, searching hyperparameters of machine learning models, and consistency analysis (PDF)
