# Supplementary material for: Accurate Prediction of Open-Circuit Voltages of Lithium-Ion Batteries via Delta Learning
Source: J Chem Theory Comput. 2025 May 15;21(10):5230–5. doi: 10.1021/acs.jctc.5c00168 (PMC12120915; doi:10.1021/acs.jctc.5c00168)
Supplement: Supplementary file 1 [file ct5c00168_si_001.pdf]

Supporting Information for
Accurate Prediction of Open Circuit Voltages of Lithium-ion Batteries via Delta-Learning

Wai Yuet Chiu[1], Chongzhi Zhang[2], Rongzhi Gao[1], Ziyang Hu[1,2,*], GuanHua Chen[1,2,*]

[1]Department of Chemistry, the University of Hong Kong, Pok Fu Lam Road, Hong Kong SAR, China, 000000

[2]Hong Kong AI Lab Limited, Pak Shek Kok, Hong Kong SAR, China, 000000

**To whom correspondence should be addressed; E-mail: hzy@yangtze.hku.hk, ghc@everest.hku.hk*

Section A. OCV-SoC Experimental Data

Table S1. A summary of all structures used in the dataset, and references to them.

Index	Structure	Reference
1	$Li_{0.5}Mn_{0.5}Ti_2(PO_4)_3$	1
2	$Li_{0.94}FePO_{3.94}N_{0.16}$	2
3	$Li_{0.958}Ni_{0.9378}Fe_{0.1042}O_2$	3
4	$Li_{0.966}Ni_{0.721}Fe_{0.1545}Co_{0.1545}O_2$	3
5	$Li_{0.96}Mn_{0.89}O_2$	4
6	$Li_{1.5}Mn_3O_4$	5
7	$Li_{1.8}Ir_{0.6}Fe_{0.6}O_3$	6
8	$Li_{2.78}O_{12}P_3V_{1.8}Zr_{0.2}$	7
9	$Li_2FeP_2O_7$	8
10	$Li_2FeP_2S_6$	9
11	Li_2FeSiO_4	10
12	$Li_2Fe(SO_4)_2$	11
13	Li_2MnO_3	12
14	$Li_2Ti_6O_{13}$	13
15	$Li_3Fe_2(PO_4)_3$	14
16	$Li_3Mn_2O_4$	5
17	$Li_5Mn_4O_9$	15
18	$Li_5V(PO_4)_2F_2$	16
19	$LiCoO_2$	17

20	$LiCoPO_4$	18
21	$LiCr_{0.05}Mn_{0.95}O_2$	19
22	$LiFe_{0.5}Mn_{0.5}PO_4$	20, 21
23	$LiFeBO_3$	22
24	$LiFeO_2$	23
25	$LiFeP_2O_7$	24
26	$LiFePO_4$	25
27	$LiFeSO_4OH$	26
28	$LiMn_2O_4$	27
29	$LiMnO_2$	28
30	$LiMnPO_4$	29
31	$LiNi_{0.33}Co_{0.33}Mn_{0.33}O_2$	30
32	$LiNi_{0.4}Co_{0.2}Mn_{0.4}O_2$	31
33	$LiNi_{0.5}Co_{0.2}Mn_{0.3}O_2$	32
34	$LiNi_{0.5}Mn_{0.15}O_4$	33
35	$LiNi_{0.6}Co_{0.2}Mn_{0.2}O_2$	34
36	$LiNi_{0.70}Co_{0.15}Al_{0.15}O_2$	35
37	$Li(Ni_{0.7}Fe_{0.15}Co_{0.15})_{1.03}O_2$	36
38	$LiNi_{0.8}Co_{0.1}Mn_{0.1}O_2$	37
39	$LiNi_{0.9}Co_{0.1}O_2$	38
40	$LiNiO_2$	39
41	$LiNiVO_4$	40
42	$LiTi_2(PO_4)_3$	41
43	$LiVOAsO_4$	42

Section B. *In-Silico* Computation of OCV-SoC Curves

The individual equilibrium states at every state of charge were modeled by MD to obtain their free energies, which were then used to compute OCV-SoC curves. The process of modeling is summarized in **Figure S1** below. Special quasi-random structure (SQS) algorithm[43] was used to remove lithium atoms from full-lithium structures.

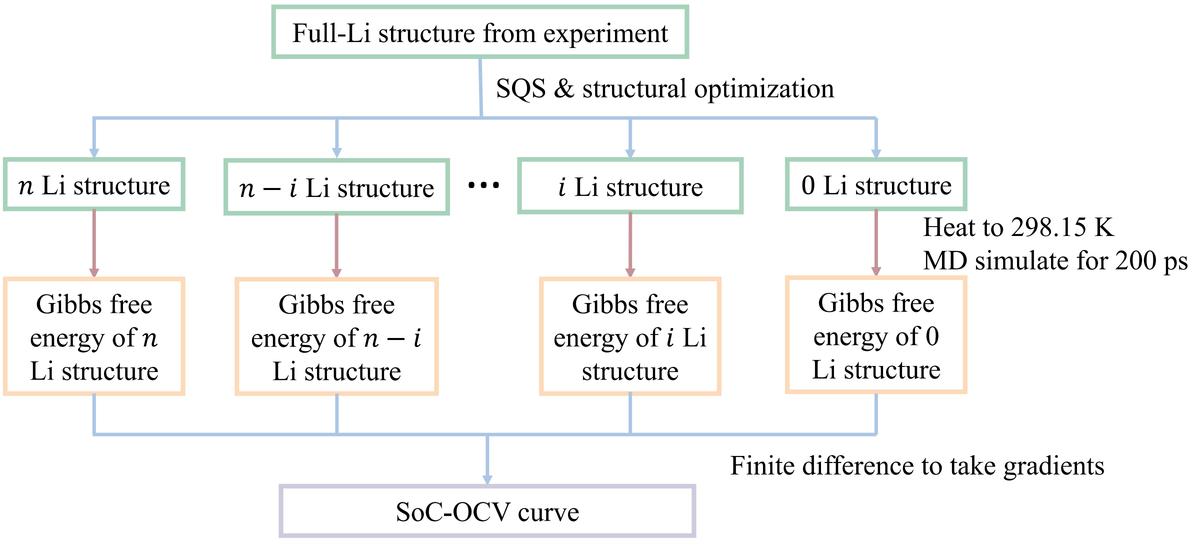

Figure S1. A flow chart of the *in-silico* computation process. The X-ray diffraction structures of the full-Li materials with n Li atoms were obtained as starting points. Then Li atoms were removed from every full-Li structure with a fixed interval i. For every delithiated structures, 20 SQS-generated structures were obtained, and the structure with lowest energy was saved for MD simulations. Upon obtaining the Gibbs free energy for each delithiated structure, gradients were computed to derive the OCV-SoC curve.

The process of delithiating a fully lithiated structure to obtain an empty lithium structure is illustrated in **Figure S2**. For example, the unit cell of $Li_3Mn_2O_4$ was first enlarged to make sure there were enough Li atoms to be removed to get around 15 to 20 delithiated structures with every two neighboring structures differed by more than 5 Li atoms. If the experiment OCV-SoC curve did not cover the full range of SoC, as also demonstrated in the **Figure S2**, only limited structures would be simulated with corresponding experimental data.

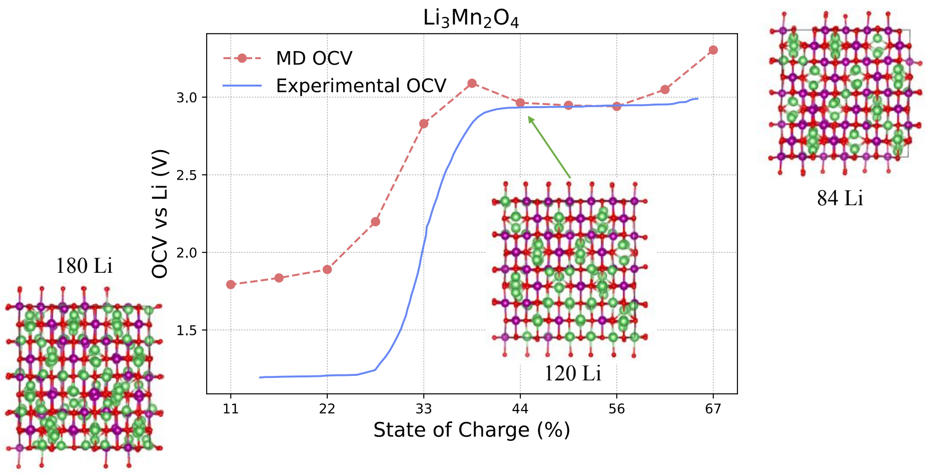

Figure S2. An example of how OCV-SoC curve of $Li_3Mn_2O_4$ was computed, where the number of Li atoms in fully lithiated structure n = 180, and the difference between adjacent structures i = 12.

The state-of-charge of simulated Li-ion batteries can be obtained by:

$$SoC = \frac{Li\ atom\ number\ in\ current\ structure}{Li\ atom\ number\ in\ fully\ lithiated\ structure}.$$

And the state-of-charge of experimentally tested Li-ion batteries can be obtained directly on graphs, if the graphs are OCV (or quasi-OCV) *vs.* SoC, or can be obtained through the equation below if only capacity is given rather than SoC:

$$SoC = \frac{capacity\ of\ current\ structure}{theoretical\ capacity\ of\ fully\ lithiated\ structure}$$

To get the Gibbs free energy, we used a machine learning interatomic potential (MLIP) with polarizable long-range interactions[44]. Every structure underwent the geometry optimization followed by a Nosé-Hoover-Parinello-Rahman NPT MD equilibration at 298.15 K and 1 atm for 200 ps, with a thermostatic characteristic time of 50 ps and a barostatic one of 500 ps. Gibbs free energies were then computed from the last 50 ps trajectories. A typical equilibration process is illustrated in **Figure S3**.

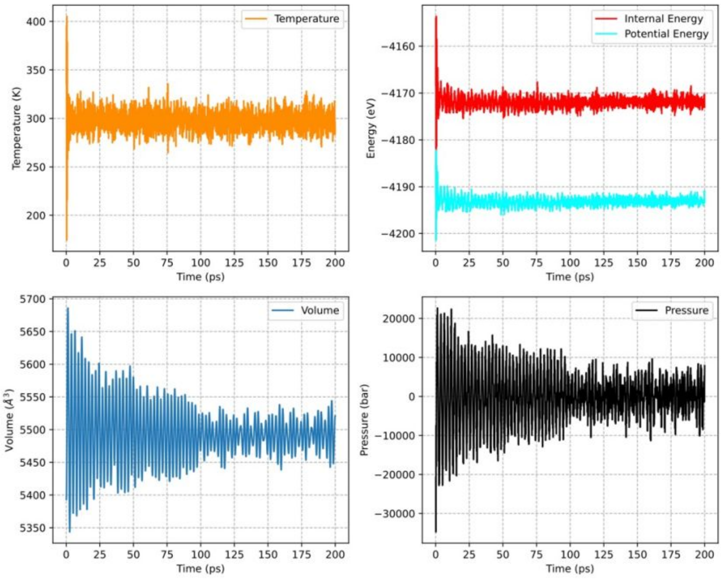

Figure S3. An example of the temperature, internal and potential energies, volume, and pressure evolution during the equilibration process. The temperature and external pressure were set to 298.15 K and 1 atm, respectively.

The OCV of a structure with *n* lithium atoms was then calculated using the following equation, where *G*(*n*) is the Gibbs energy of it, *E*(Li) is the per atom energy of bulk *bcc* lithium, and *e* is the elementary charge.

$$\mathrm{OCV}(n) = -\frac{1}{e}\left[\frac{\partial G(n)}{\partial n} - E(\mathrm{Li})\right].$$

Section C. Benchmarking on Machine Learning Interatomic Potential

To demonstrate the capability of our MLIP of describing delithiated structures, we benchmark the energy differences between delithiated and fully lithiated $LiFePO_4$ structures calculated by our MLIP against those obtained using the PBE+U methodology:

$$E_{\mathrm{diff,\ Li}_x\mathrm{Fe}_4\mathrm{P}_4\mathrm{O}_{16}} = \frac{\left(E_{\mathrm{Li}_x\mathrm{Fe}_4\mathrm{P}_4\mathrm{O}_{16}} - E_{\mathrm{Li}_4\mathrm{Fe}_4\mathrm{P}_4\mathrm{O}_{16}}\right)}{N_{\mathrm{atoms,Li}_x\mathrm{Fe}_4\mathrm{P}_4\mathrm{O}_{16}}}.$$

The results are listed in **Table S2**.

Table S2. Single-point energy (in eV/atom) for Pnma-LiFePO4 (mp-19017) calculated by the PBE+U method ($U-J$ = 5.3 eV for Fe) and our MLIP.

	$E_{\mathrm{diff,\ Li}_3\mathrm{Fe}_4\mathrm{P}_4\mathrm{O}_{16}}$	$E_{\mathrm{diff,\ Li}_2\mathrm{Fe}_4\mathrm{P}_4\mathrm{O}_{16}}$	$E_{\mathrm{diff,\ Li}_1\mathrm{Fe}_4\mathrm{P}_4\mathrm{O}_{16}}$
PBE+U	0.163	0.429	0.702
Our model	0.196	0.441	0.668
Difference	−0.033	−0.012	0.034

Section D. Feature Importance Analysis

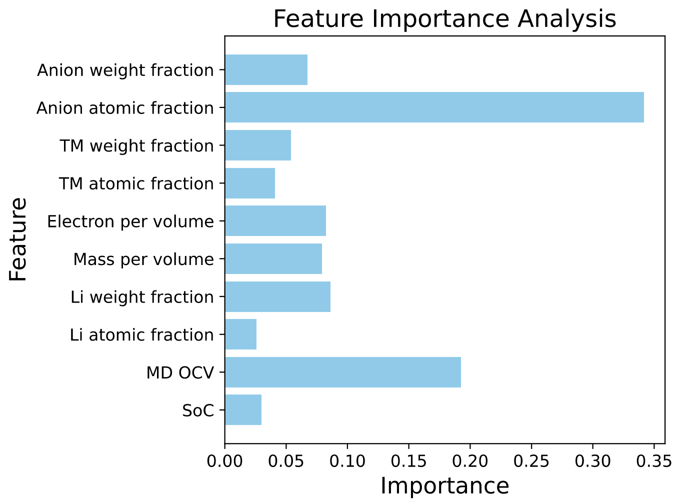

Figure S4. Feature importance analysis for the trained random forest model. Anion atomic fraction and MD simulated OCV are two of the most important features for training.

Section E. Search Space for Machine Learning Models

Table S3. Search spaces for training RF, SVR, GB, and XGBoost with Bayesian optimization.

Search space \ Model	RF	SVR	GB	XGBoost
n_estimators	Integer(400, 600)	N/A	Integer(400, 600)	Integer(900, 1300)
max_depth	Integer(40, 60)	N/A	Integer(1, 10)	Integer(8, 17)
min_samples_split	Integer(2, 10)	N/A	Integer(2, 7)	Integer(2, 10)
min_samples_leaf	Integer(1, 10)	N/A	Integer(2, 7)	Integer(1, 7)
learning_rate	N/A	N/A	Real(0.008, 0.02)	Real(0.001, 0.1)
loss	N/A	N/A	N/A	N/A
C	N/A	Real(1e-3, 1e+3, prior='log-uniform')	N/A	N/A
degree	N/A	Integer(1, 8)	N/A	N/A
epsilon	N/A	Real(1e-6, 1e+1, prior='log-uniform')	N/A	N/A
gamma	N/A	Real(1e-6, 1e+1, prior='log-uniform')	N/A	N/A
kernel	N/A	Categorical(['linear', 'poly', 'rbf'])	N/A	N/A

Section F. Consistency Test

Two trials on $Li_{0.96}Mn_{0.89}O_2$ of the whole process including SQS generation of delithiation process, MD simulation, and machine learning prediction, are compared to evaluate the randomness involved in the computation of SoC-OCV curves.

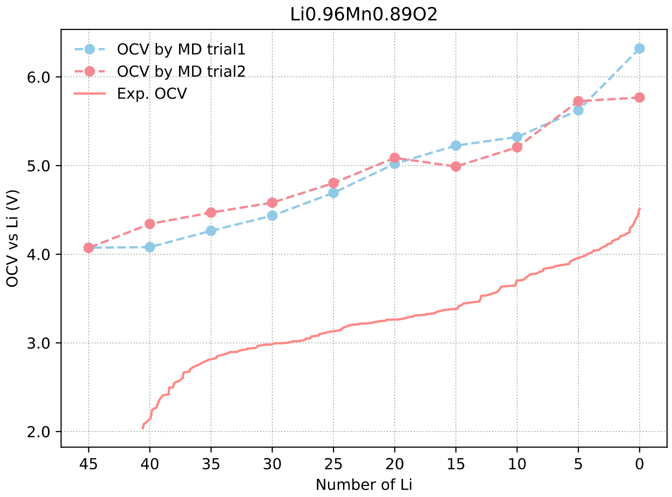

Figure S5. Comparison of the MD SoC-OCV curves generated from two independent trials. The MAE between them is 0.054 V.

Taking the two MD-simulated OCV-SoC curves as input for the trained random forest model, the resulting MAE is 0.019 V, much lower than the training MAE 0.148 V, demonstrating that the effect of randomness would be slight.

(1) Aatiq, A.; Ménétrier, M.; El Jazouli, A.; Delmas, C. Structural and lithium intercalation studies of Mn (0.5− x) CaxTi2 (PO4) 3 phases (0≤ x≤ 0.50). *Solid State Ionics* **2002**, *150* (3-4), 391-405.

(2) Mayer, S. F.; de la Calle, C.; Fernández-Díaz, M. T.; Amarilla, J. M.; Alonso, J. A. Nitridation effect on lithium iron phosphate cathode for rechargeable batteries. *RSC advances* **2022**, *12* (6), 3696-3707.

(3) Prado, G.; Fournes, L.; Delmas, C. Mixed cobalt and iron substituted lithium nickelate: a structural and electrochemical study. *Solid State Ionics* **2000**, *138* (1-2), 19-30.

(4) Raekelboom, E.; Hector, A.; Owen, J.; Vitins, G.; Weller, M. Syntheses, structures, and preliminary electrochemistry of the layered lithium and sodium manganese (IV) oxides, A2Mn3O7. *Chemistry of materials* **2001**, *13* (12), 4618-4623.

(5) Thackeray, M. M.; David, W. I.; Bruce, P. G.; Goodenough, J. B. Lithium insertion into manganese spinels. *Materials research bulletin* **1983**, *18* (4), 461-472.

(6) Kobayashi, H.; Uebou, Y.; Tabuchi, M.; Kageyama, H.; Yamamoto, Y.; Matsuoka, M.; Tamaki, J. Structure, physical properties, and charge-discharge characteristics of Fe-doped Li2IrO3. *Journal of the Electrochemical Society* **2003**, *150* (11), A1408.

(7) Sato, M.; Ohkawa, H.; Yoshida, K.; Saito, M.; Uematsu, K.; Toda, K. Enhancement of discharge capacity of Li3V2 (PO4) 3 by stabilizing the orthorhombic phase at room temperature. *Solid State Ionics* **2000**, *135* (1-4), 137-142.

(8) Ye, T.; Barpanda, P.; Nishimura, S.-i.; Furuta, N.; Chung, S.-C.; Yamada, A. General observation of Fe3+/Fe2+ redox couple close to 4 V in partially substituted Li2FeP2O7 pyrophosphate solid-solution cathodes. *Chemistry of Materials* **2013**, *25* (18), 3623-3629.

(9) Takada, K.; Michiue, Y.; Inada, T.; Kajiyama, A.; Kouguchi, M.; Kondo, S.; Watanabe, M.; Tabuchi, M. Lithium iron thio-phosphate: a new 3 V sulfide cathode. *Solid State Ionics* **2003**, *159* (3-4), 257-263.

(10) Yabuuchi, N.; Yamakawa, Y.; Yoshii, K.; Komaba, S. Low-temperature phase of Li 2 FeSiO 4: crystal structure and a preliminary study of electrochemical behavior. *Dalton transactions* **2011**, *40* (9), 1846-1848.
(11) Lander, L.; Reynaud, M.; Rousse, G.; Sougrati, M. T.; Laberty-Robert, C.; Messinger, R. J.; Deschamps, M.; Tarascon, J.-M. Synthesis and electrochemical performance of the orthorhombic Li2Fe (SO4) 2 polymorph for Li-ion batteries. *Chemistry of Materials* **2014**, *26* (14), 4178-4189.
(12) Xu, J.; Kaufman, L.; Robles Hernández, F. C.; Pramanik, A.; Babu, G.; Nanda, J.; McCloskey, B. D.; Ajayan, P. M. Effects of Ball Milling on the Electrochemical Capacity and Interfacial Stability of Li2MnO3 Cathode Materials. *ACS Applied Energy Materials* **2023**, *6* (9), 5026-5036.
(13) Kataoka, K.; Awaka, J.; Kijima, N.; Hayakawa, H.; Ohshima, K.-i.; Akimoto, J. Ion-exchange synthesis, crystal structure, and electrochemical properties of Li2Ti6O13. *Chemistry of Materials* **2011**, *23* (9), 2344-2352.
(14) Padhi, A.; Nanjundaswamy, K.; Masquelier, C.; Okada, S.; Goodenough, J. Effect of structure on the Fe3+/Fe2+ redox couple in iron phosphates. *Journal of the Electrochemical Society* **1997**, *144* (5), 1609.
(15) De Kock, A.; Rossouw, M.; De Picciotto, L.; Thackeray, M.; David, W.; Ibberson, R. Defect spinels in the system Li2O. yMnO2 (y> 2.5): A neutron-diffraction study and electrochemical characterization of Li2Mn4O9. *Materials research bulletin* **1990**, *25* (5), 657-664.
(16) Yin, S.-C.; Herle, P. S.; Higgins, A.; Taylor, N.; Makimura, Y.; Nazar, L. Dimensional reduction: Synthesis and structure of layered Li5M (PO4) 2F2 (M= V, Cr). *Chemistry of materials* **2006**, *18* (7), 1745-1752.
(17) Wang, Z.; Wang, Z.; Peng, W.; Guo, H.; Li, X.; Wang, J.; Qi, A. Structure and electrochemical performance of LiCoO 2 cathode material in different voltage ranges. *Ionics* **2014**, *20*, 1525-1534.
(18) YeſildaL, A.; Can, M.; Aktürk, S. A practical and effective strategy for the thin and uniform carbon layer onto LiCoPO4 cathode surface in terms of the rate capability and cycle stability. *Materials Research Bulletin* **2016**, *83*, 1-11.
(19) Ammundsen, B.; Desilvestro, J.; Groutso, T.; Hassell, D.; Metson, J.; Regan, E.; Steiner, R.; Pickering, P. Formation and structural properties of layered LiMnO2 cathode materials. *Journal of the Electrochemical Society* **2000**, *147* (11), 4078.
(20) Li, X.; Zhang, B.; Zhang, Z.; He, L.; Li, H.; Huang, X.; Wang, F. Crystallographic structure of LiFe1− xMnxPO4 solid solutions studied by neutron powder diffraction. *Powder Diffraction* **2014**, *29* (3), 248-253.
(21) Ding, J.; Su, Z.; Tian, H. Synthesis of high rate performance LiFe1− xMnxPO4/C composites for lithium-ion batteries. *Ceramics International* **2016**, *42* (10), 12435-12440.
(22) Dong, Y.; Zhao, Y.; Shi, Z.; An, X.; Fu, P.; Chen, L. The structure and electrochemical performance of LiFeBO3 as a novel Li-battery cathode material. *Electrochimica Acta* **2008**, *53* (5), 2339-2345.
(23) Hirayama, M.; Tomita, H.; Kubota, K.; Kanno, R. Structure and electrode reactions of layered rocksalt LiFeO2 nanoparticles for lithium battery cathode. *Journal of Power Sources* **2011**, *196* (16), 6809-6814.
(24) Kim, H.; Lee, S.; Park, Y.-U.; Kim, H.; Kim, J.; Jeon, S.; Kang, K. Neutron and X-ray diffraction study of pyrophosphate-based Li2–x MP2O7 (M= Fe, Co) for lithium rechargeable battery electrodes. *Chemistry of Materials* **2011**, *23* (17), 3930-3937.
(25) Zhao, B.; Jiang, Y.; Zhang, H.; Tao, H.; Zhong, M.; Jiao, Z. Morphology and electrical properties of carbon coated LiFePO4 cathode materials. *Journal of Power Sources* **2009**, *189* (1), 462-466.

(26) Subban, C. V.; Ati, M.; Rousse, G.; Abakumov, A. M.; Van Tendeloo, G.; Janot, R.; Tarascon, J.-M. Preparation, structure, and electrochemistry of layered polyanionic hydroxysulfates: LiMSO4OH (M= Fe, Co, Mn) electrodes for Li-ion batteries. *Journal of the American Chemical Society* **2013**, *135* (9), 3653-3661.
(27) Sigala, C.; Verbaere, A.; Mansot, J.; Guyomard, D.; Piffard, Y.; Tournoux, M. The Cr-substituted spinel Mn oxides LiCryMn2− yO4 (0≤ y≤ 1): Rietveld analysis of the structure modifications induced by the electrochemical lithium deintercalation. *Journal of Solid State Chemistry* **1997**, *132* (2), 372-381.
(28) Armstrong, A. R.; Bruce, P. G. Synthesis of layered LiMnO2 as an electrode for rechargeable lithium batteries. *Nature* **1996**, *381* (6582), 499-500.
(29) Oh, S. M.; Oh, S. W.; Yoon, C. S.; Scrosati, B.; Amine, K.; Sun, Y. K. High-performance carbon-LiMnPO4 nanocomposite cathode for lithium batteries. *Advanced Functional Materials* **2010**, *20* (19), 3260-3265.
(30) Xu, Z.; Xiao, L.; Wang, F.; Wu, K.; Zhao, L.; Li, M.-R.; Zhang, H.-L.; Wu, Q.; Wang, J. Effects of precursor, synthesis time and synthesis temperature on the physical and electrochemical properties of Li (Ni1− x− yCoxMny) O2 cathode materials. *Journal of Power Sources* **2014**, *248*, 180-189.
(31) Chen, Z.; Chao, D.; Liu, J.; Copley, M.; Lin, J.; Shen, Z.; Kim, G.-T.; Passerini, S. 1D nanobar-like LiNi 0.4 Co 0.2 Mn 0.4 O 2 as a stable cathode material for lithium-ion batteries with superior long-term capacity retention and high rate capability. *Journal of Materials Chemistry A* **2017**, *5* (30), 15669-15675.
(32) Wu, K.; Wang, F.; Gao, L.; Li, M.-R.; Xiao, L.; Zhao, L.; Hu, S.; Wang, X.; Xu, Z.; Wu, Q. Effect of precursor and synthesis temperature on the structural and electrochemical properties of Li (Ni0. 5Co0. 2Mn0. 3) O2. *Electrochimica Acta* **2012**, *75*, 393-398.
(33) Patoux, S.; Sannier, L.; Lignier, H.; Reynier, Y.; Bourbon, C.; Jouanneau, S.; Le Cras, F.; Martinet, S. High voltage nickel manganese spinel oxides for Li-ion batteries. *Electrochimica Acta* **2008**, *53* (12), 4137-4145.
(34) Xu, L.; Zhou, F.; Kong, J.; Chen, Z.; Chen, K. Synthesis of Li (Ni 0.6 Co 0.2 Mn 0.2) O 2 with sodium DL-lactate as an eco-friendly chelating agent and its electrochemical performances for lithium-ion batteries. *Ionics* **2018**, *24*, 2261-2273.
(35) Guilmard, M.; Pouillerie, C.; Croguennec, L.; Delmas, C. Structural and electrochemical properties of LiNi0. 70Co0. 15Al0. 15O2. *Solid State Ionics* **2003**, *160* (1-2), 39-50.
(36) Prado, G.; Fournes, L.; Delmas, C. On the LixNi0. 70Fe0. 15Co0. 15O2 system: An X-ray diffraction and Mössbauer study. *Journal of Solid State Chemistry* **2001**, *159* (1), 103-112.
(37) Li, J.; Li, Y.; Yi, W.; Ma, P. Improved electrochemical performance of cathode material LiNi 0.8 Co 0.1 Mn 0.1 O 2 by doping magnesium via co-precipitation method. *Journal of Materials Science: Materials in Electronics* **2019**, *30*, 7490-7496.
(38) Park, H. W.; Hwang, J. U.; Im, J. S.; Lee, J. D. Electrochemical properties of LiNi0. 9Co0. 1O2 cathode material prepared by co-precipitation using an eco-friendly chelating agent. *Journal of Solid State Electrochemistry* **2022**, *26* (8), 1567-1576.
(39) Arai, H.; Tsuda, M.; Sakurai, Y. Lithium nickelate electrodes with enhanced high-temperature performance and thermal stability. *Journal of power sources* **2000**, *90* (1), 76-81.
(40) Chitra, S.; Kalyani, P.; Yebka, B.; Mohan, T.; Haro-Poniatowski, E.; Gangadharan, R.; Julien, C. Synthesis, characterization and electrochemical studies of LiNiVO4 cathode material in rechargeable lithium batteries. *Materials chemistry and physics* **2000**, *65* (1), 32-37.
(41) Luo, J.-Y.; Chen, L.-J.; Zhao, Y.-J.; He, P.; Xia, Y.-Y. The effect of oxygen vacancies on the structure and electrochemistry of LiTi2 (PO4) 3 for lithium-ion batteries: A combined experimental and theoretical study. *Journal of Power Sources* **2009**, *194* (2), 1075-1080.

(42) Gaubicher, J.; Orsini, F.; Le Mercier, T.; Llorente, S.; Villesuzanne, A.; Angenault, J.; Quarton, M. Synthesis, structure, and physical studies of the new β-LiVOAsO4 compound. *Journal of Solid State Chemistry* **2000**, *150* (2), 250-257.
(43) Zunger, A.; Wei, S.-H.; Ferreira, L.; Bernard, J. E. Special quasirandom structures. *Physical review letters* **1990**, *65* (3), 353.
(44) Gao, R.; Yam, C.; Mao, J.; Chen, S.; Chen, G.; Hu, Z. Enhancing universal machine learning potentials with polarizable long-range interactions. *arXiv preprint arXiv:2410.13820* **2024**.
